# Thrombin Generation in Preterm Newborns With Intestinal Failure-Associated Liver Disease

**DOI:** 10.3389/fped.2020.00510

**Published:** 2020-08-26

**Authors:** Stefano Ghirardello, Genny Raffaeli, Erica Scalambrino, Valeria Cortesi, Paola Roggero, Flora Peyvandi, Fabio Mosca, Armando Tripodi

**Affiliations:** ^1^Fondazione IRCCS Ca' Granda Ospedale Maggiore Policlinico, NICU, Milan, Italy; ^2^Department of Clinical Sciences and Community Health, Università degli Studi di Milano, Milan, Italy; ^3^Fondazione IRCCS Ca' Granda Ospedale Maggiore Policlinico, Angelo Bianchi Bonomi Hemophilia and Thrombosis Center and Fondazione Luigi Villa, Milan, Italy; ^4^Department of Pathophysiology and Transplantation, Università degli Studi di Milano, Milan, Italy

**Keywords:** global coagulation assay, hemostasis, prematurity, parenteral nutrition, vitamin K, cholestasis, thrombosis

## Abstract

**Background and Aim:** Intestinal failure-associated liver disease (IFALD) affects one-fifth of neonates receiving parenteral nutrition (PN) for more than 2 weeks. We aimed to define the effect of IFALD on hemostasis of preterm infants.

**Methods:** This is an ancillary analysis of a prospective study aimed at defining coagulation in preterm infants. We included neonates exposed to PN (at least 14 days), in full-enteral feeding. We compared thrombin generation in the presence of thrombomodulin, defined as endogenous thrombin potential-ETP, PT, aPTT between infants with IFALD vs. those without (controls), at birth, and after 30 days. IFALD was defined as conjugated bilirubin ≥1 mg/dl.

**Results:** We enrolled 92 preterm infants (32 IFALD; 60 controls). Cholestatic patients had a lower birthweight, longer exposure to PN, and longer hospitalization. Infants with IFALD showed longer median PT (12.8-vs.-12 sec; *p* = 0.02) and aPTT (39.2-vs.-36.5 sec; *p* = 0.04) than controls, with no difference in ETP.

**Conclusions:** Despite prolonged PTs and aPTTs infants with IFALD had similar ETP than those without.

## Introduction

Parenteral nutrition (PN) provides adequate nutrients to very preterm infants in the first weeks of life, when tolerance to increasing amounts of enteral nutrition is limited. Despite evident benefits, PN for more than 2 weeks may cause liver damage, of which intestinal failure-associated liver disease (IFALD) is the most common manifestation (1).

The incidence of IFALD is around 28% in infants receiving PN for more than 14 days (1–3). In cholestasis, reduced bile flow in the intestinal lumen may impair the absorption of fat-soluble vitamins. Among others, vitamin K (VK) is essential for the ⋎-carboxylation of VK-dependent pro- and anticoagulant factors (4).

Prophylactic VK given at birth and during PN prevents preterm newborns from VK deficiency (5). If IFALD develops, full-enterally fed newborns may experience VK deficiency due to reduced intestinal absorption; in these patients, oral or intramuscular VK supplementation is suggested to reduce the bleeding risk and correct possible VK deficiency (6, 7).

Standard coagulation tests have relatively poor sensitivity and specificity to identify subclinical VK deficiency and assess bleeding risk in newborns with IFALD (4). Indeed, prothrombin time (PT) is not recommended for the evaluation of subclinical VK deficiency. Recently, international normalized ratio (INR) was found to be a poor marker of VK status in infants affected by short bowel syndrome and IFALD (8). A partially carboxylated prothrombin induced by VK absence (PIVKA-II) is considered a more sensitive indicator of subclinical VK deficiency; however, PIVKA-II analysis is uncommon in clinical practice (9, 10).

Moreover, both PT and PIVKA-II account for the procoagulant factors but much less for the anticoagulant counterpart, and are therefore not truly representative of the whole hemostatic process occurring *in vivo*.

Indeed, during the neonatal period, coagulation is rebalanced because of the concomitant deficiency of pro- and anticoagulant drivers. Compared to term newborns, preterm infants express a procoagulant imbalance, as assessed by thrombin generation procedures (11).

Thrombin generation procedures can account for the net amount of thrombin that any given plasma can potentially generate, based on the balance between pro- and anticoagulant drivers.

We hypothesized that, in patients with IFALD, subclinical VK deficiency might affect thrombin generation. To address this issue, we compared preterm newborns with and without IFALD.

## Materials and Methods

This study is a secondary analysis of a prospective observational study conducted at the Neonatal Intensive Care Unit (NICU) Fondazione IRCCS Ca' Granda Ospedale Maggiore Policlinico (07/2015-06/2018) that aimed at defining the hemostatic profile in very low birth weight infants in the first month of life (11, 12). Inclusion and exclusion criteria and demographic data of the original cohort were detailed elsewhere (11, 12). The Institutional Review Board approved the study protocol, and the written informed consent was obtained from patients' tutors. All procedures were carried out following the Helsinki Declaration.

### Study Population

In this study, we included all patients from the original cohort exposed to PN and for whom thrombin generation was available both at birth and at 30 days of life.

Exclusion criteria were: (i) Extra-dose(s) of oral or intramuscular VK before blood sampling. (ii) Plasma transfusion before blood sampling, and (iii) Causes of cholestatic jaundice other than IFALD.

Patients were daily evaluated for the occurrence of sepsis, bleeding (intraventricular, pulmonary, gastrointestinal bleeding), platelet, or plasma transfusion. Demographic data included birthweight (adequate vs. small for gestational age [SGA]), delivery mode, Apgar score, Clinical Risk Index for Babies (CRIB-II score), conjugated bilirubin, feeding mode, days of PN or enteral feeding, use of lipid emulsion supplements for human milk fortification, bowel incontinuity, septic events, need for antimicrobial therapy and probiotic use.

As per standard institutional protocol, all neonates exposed to PN received Smoflipid emulsion, which contains fish oil-derived omega-3 and medium chain triglycerides. In our NICU, the individualized human milk fortification is current practice for all neonates with a birthweight ≤ 1,800 grams receiving an enteral intake of 80 ml/kg/die, being human milk more than the half. The administration of single/multi-nutrients fortifiers or supplements of lipids, proteins and/or carbohydrates over the entire NICU stay is modulated by means of serial milk analyses to achieve a targeted human milk fortification. Enterally-fed infants received probiotics, lactoferrin and supplements of vitamin A and D.

IFALD was defined based on the value of conjugated bilirubin ≥1 mg/dl in patients with at least 14 days of PN. SGA neonates were those with birthweight below the 10th centile for GA, according to the Fenton growth charts for preterm infants.

The cohort was divided based on the presence (study group) or absence (control group) of IFALD.

### Blood Collection and Measurements

Blood samples were collected within 72 h from birth and at 30 days of life from direct venipuncture in EDTA or citrated tubes (3.2% trisodium citrate; 9:1 vol/vol ratio). Laboratory procedures included thrombin generation, PT, activated partial thromboplastin time (APTT), fibrinogen, full blood cell count, total and direct serum bilirubin.

### Thrombin Generation Procedure

Thrombin generation was assessed with a homemade method, as previously described (13). Briefly, coagulation was initiated in platelet-poor plasma by the addition of tissue factor (1 pM) (Recombiplastin 2G, Werfen Orangeburg, NY), synthetic phospholipids (1 μM) as triggers, and soluble rabbit thrombomodulin (4 nM) (Hematology Technologies, Essex, VT) as protein C (PC) activator (14). Thrombin generation was monitored and recorded by a dedicated software that calculates the area under the thrombin generation curve, defined as endogenous thrombin potential (ETP), expressed as nMxminutes.

### Statistical Analyses

We compared demographic characteristics, full blood cell count, standard coagulation tests, ETP at birth, and 30 days of life in patients with PNAC and the control group. We then compared patients with and without IFALD, both in full-enteral feeding for at least 15 days after PN discontinuation.

We used the two-sided Mann–Whitney *U*-test for comparison between continuous non-normal distributed variables, Wilcoxon test for quantitative variables (non-parametric paired data), and the χ2-test for categorical variables. Results were reported as median and range (min-max). Analyses were performed with the SPSS statistical package (SPSS, Illinois).

## Results

We included 92 infants, 32 of which affected by IFALD. Table 1 shows demographic characteristics and coagulation profile at birth and 30 days of life. Overall, four patients presented with bowel incontinuity, due to surgical necrotizing enterocolits. Compared to the control group, patients with IFALD had lower birth weight, higher incidence of growth restriction, higher CRIB scores, longer duration of PN, and hospital stay (Table 1). Although the number of septic events was similar between groups, the exposure to antimicrobial therapy was higher in the IFALD group (Table 1).

**Table 1:** Demographics and coagulation profile at birth (1) and at 30 days of life (2) of the study population.

	**NO-IFALD**		**IFALD**		
	***N***		***N***		***P*-value**
**DEMOGRAPHIC DATA**					
GA (weeks)^*^	60	30 (24–34)	32	29 (25–35)	ns
PCA at sample time (weeks)^*^	60	34 (28–39)	32	35 (30–38)	ns
BW (grams)^*^	60	1,275 (700–1,495)	32	1,065 (660–1,490)	**0.001**
SGA^§^	60	8 (13.3)	32	13 (40.6)	**0.003**
Gender male^§^	60	24 (40)	32	16 (50)	ns
Cesarean section^§^	60	54 (90)	32	26 (81.3)	ns
Bowel incontinuity^§^	60	1 (2)	32	3 (9)	ns
Death^§^	60	0 (0)	32	1 (3.1)	ns
Lenght of stay (days)^*^	60	54 (27–270)	32	75 (30–269)	**0.005**
PN (days)^*^	60	21 (6–49)	32	34 (14–179)	**0.000**
Singleton^§^	60	25 (41.7)	32	17 (53.1)	ns
CRIB II^*^	60	6 (2–16)	32	8 (3–14)	**0.018**
Apgar 5 min^*^	59	8 (6–10)	31	9 (6–10)	ns
Time free from PN (days)^*^	44	12 (1–37)	32	4 (0–32)	**0.002**
Septic events^*^	60	0 (0–3)	32	1 (0–6)	ns
Antimicrobial therapy (days)^*^	60	7 (3–75)	32	10 (4–93)	**0.04**
**HEMOSTATIC PROFILE**					
PT (s) at birth^*^	42	13.5 (10.7–25.9)	23	14.8 (10.4–51.2)	**0.036**
PT (s) at 30 days^*^	57	12 (9.7–16.6)	32	12.8 (10.4–16.3)	**0.02**
*P*-value		**<0.001**		**0.001**	
APTT (s) at birth	41	49.3 (30.8–97.5)	22	58 (28.4–86.4)	**0.013**
APTT (s) at 30 days	57	36.5 (26.1–58.2)	32	39.2 (28.3–59.9)	**0.041**
*P*-value		<0.001		<0.001	
Fibrinogen (mg/dl) at birth^*^	45	296 (79–550)	24	169 (41–409)	**0.016**
Fibrinogen (mg/dl) at 30 days^*^	60	229 (103–393)	32	223 (117–442)	ns
*P*-value		0.337		0.113	
*P*-value		0.221		0.287	
ETP_TM (nMol/l × min) at birth	49	1223.5 (417.5–2141.5)	25	1,213.5 (188–1,715)	ns
ETP_TM (nMol/l × min) at 30 days^*^	57	1108.5 (444–1,905)	29	1007.5 (329.5–1,829)	ns
*P*-value		**0.262**		**0.639**	
Hct (%)at birth	57	49 (26–65)	31	44 (30–61)	ns
Hct (%)at 30 days^*^	56	31(24–39)	31	31 (24–39)	ns
*P*-value		**<0.001**		**<0.001**	
Platelet count (10^3^/μl) at birth	57	226 (30–433)	31	184 (58–352)	**0.002**
Platelet count(10^3^/μl) at 30 days^*^	56	443 (98–853)	31	318 (45–581)	**<0.001**
*P*-value		**<0.001**		**<0.001**	

Comparison of demographic and hemostatic characteristics at birth and at 30 days of life within each group and between groups (IFALD vs. no IFALD). Bold values P < 0.05 were considered as statistically significant. APTT, activated partial thromboplastin time; BW, birth weight; CRIB II, clinical risk index babies score; ETP_TM, endogenous thrombin potential, with thrombomodulin; GA, gestational age; IFALD, intestinal failure-associated liver disease; PCA, post-conceptional age; PT, prothrombin time; SGA, small for gestational age; PN, parenteral nutrition. Ns, not significant.

* Results expressed as median (minimum-maximum).

§ Results expressed as number (%).

*U-Mann–Whitney test was used for quantitative variables (non-parametric non-paired data); Wilcoxon test was used for quantitative variables (non-parametric paired data); Chi-squared test was used for categorical variables*.

PTs and aPTTs were shorter at 30 days than at birth regardless of IFALD (p < 0.001), but were prolonged in the IFALD group both at birth and after 30 days (*p* < 0.05). Conversely, we found no differences in the ETP neither at birth nor after 30 days of life in the group with or without IFALD.

Table 2 reports demographic characteristics and coagulation profiles of patients with and without IFALD after PN discontinuation and fifteen days of full enteral feeding. The two groups shared similar clinical and hemostatic profiles.

**Table 2:** Demographics and coagulation profile of patients with ≥ 15 days of full enteral feeding.

	**NO–IFALD**		**IFALD**		
	***N***		***N***		***P*-value**
**DEMOGRAPHIC DATA**					
GA (weeks)^*^	18	30 (27–34)	10	30 (26–33)	ns
PCA at sample time (weeks)^*^	18	36 (33–39)	10	36 (33–37)	ns
BW (grams)^*^	18	1,250 (800–1,495)	10	1,173 (840–1,330)	ns
SGA^§^	18	4 (22.2)	10	4 (40)	ns
Gender male^§^	18	6 (33.3)	10	7 (70)	ns
Cesarean Section^§^	18	18 (100)	10	7 (70)	**0.014**
Death^§^	18	0 (0)	10	0 (0)	–
Lenght of stay (days)^*^	18	48 (37–105)	10	54 (30–111)	ns
PN (days)^*^	18	20 (12–27)	10	21 (14–35)	ns
Singleton^§^	18	6 (33.3)	10	7 (70)	ns
CRIB II^*^	18	6 (2–9)	10	7 (4–12)	ns
Apgar 5 min^*^	18	8 (6–10)	10	8 (6–10)	ns
Time free from PN (days)^*^	18	20 (15–37)	10	16 (15–32)	ns
Fortified Human milk^§^	18	15 (83.3)	10	9 (90)	ns
Supplements of lipids for human milk fortification(days)^*^	18	23(0–78)	10	34(0–74)	ns
**HEMOSTATIC PROFILE**					
PT (s)^*^	17	12 (10.3–13.5)	10	11.7 (10.4–14.9)	ns
APTT (s)^*^	17	35.5 (27.9–51.5)	10	37.8 (33.7–59.9)	ns
Fibrinogen (mg/dl)^*^;$$	18	219 (146–393)	10	216 (117–266)	ns
LT (min)^*^	18	4.66 (2.95–5.92)	10	4.55 (4.08–7.77)	ns
ETP_TM (nMol/l × min)^*^	16	1053.5 (492.5–1373.5)	10	967 (507–1612)	ns
Hct (%)^*^	17	32.1 (24.2–38.9)	10	31.9 (27.2–37.1)	ns
Platelet count (10^3^/μL)^*^	17	448 (264–853)	10	389 (131–581)	ns
Total bilirubin (mg/dl)^*^	18	1.86 (0.65–9.51)	10	4.14 (1.84–10.56)	**0.031**
Direct bilirubin (mg/dl)^*^	13	0.67 (0.33–1)	10	1.98 (1.24–4.46)	**0.000**

Bold values P < 0.05 were considered as statistically significant; APTT, activated partial thromboplastin time; BW, birth weight; CRIB II, clinical risk index babies score; ETP_TM, endogenous thrombin potential, with thrombomodulin; GA, gestational age; IFALD, intestinal failure-associated liver disease; PCA, post-conceptional age; PT, prothrombin time; SGA, small for gestational age; PN, parenteral nutrition; ns, not significant.

* Results expressed as median (minimum-maximum).

§ Results expressed as number (%).

*U-Mann-Whitney test was used for quantitative variables (non-parametric non-paired data); Chi-squared test was used for categorical variables*.

## Discussion

Our study showed that the coagulation profile assessed by ETP at birth or after 1 month of life was similar between preterm newborns with IFALD compared to those without IFALD.

In contrast, the cohort of patients with IFALD had longer PTs and aPTTs compared to the control group both at birth and at 1 month of age. These results indicate that PT/aPTT and ETP respond differently to the same coagulation alteration. This conclusion is in line with that derived from studies on PT/aPTT or ETP when used in the neonatal setting (15, 16), and further indicate that PT/aPTT do not truly represent the real situation occurring *in vivo*. Obviously, there might be other explanations, such as the relatively small number of patients enrolled in the IFALD group, compared to healthy preterm infants. Furthermore, we cannot exclude that the more severe clinical conditions of IFALD patients at birth, as supported by their higher CRIB score and the higher incidence of intrauterine growth restriction, may have affected results (12, 17, 18). Moreover, the higher exposure to antimicrobial drugs may have contributed to alter intestinal flora and subsequent vitamin K synthesis in the IFALD group. Indeed menaquinones, which represent the major form (more than 50%) of human VK storage, are produced by colonic bacteria (19).

Interestingly, in the subgroup of exclusively enterally-fed patients, mostly with fortified human milk, we observed no differences in ETP between IFALD patients and the control group.

Cholestatic newborns are at risk for acquired coagulopathy due to reduced VK absorption (20) and limited tissue reserves that are rapidly catabolized, with 60–70% of a single VK dose being excreted in about 3 days (4). Indeed, oral or parenteral VK supplementation is suggested in full enterally-fed preterm newborns with IFALD, although effective VK intestinal absorption in these patients is unknown.

VK promotes the γ-carboxylation of VK pro- and anticoagulant factors (e.g., factor II, VII, IX, X, protein C, and S). When subclinical VK deficiency occurs, inadequate carboxylation of factor II is manifest by the release into the circulation of a functionally deficient abnormal factor II protein, called PIVKA-II (4).

PIVKA-II and serum VK measurements are considered the most sensitive markers of VK status (4). In contrast, the PT is a late indicator of VK deficiency as it could be normal even when factor II is reduced to half of the normal value (4, 9, 10). However, PT, PT-INR, and PIVKA-II hardly predict the bleeding risk in cholestatic patients because they account for the action of procoagulant factors only. In this respect, Dao et al. recently reported trends of INR and fecal excretion of VK in a small group of infants with small bowel syndrome and IFALD. They demonstrated that, during cholestasis reversal, INR remained unchanged and did not correlate with the increased daily fecal excretion of VK. Although a direct comparison with our results is not feasible due to the differences in the study population, this article further supports the fact that INR cannot be used alone to monitor VK status (8).

Another recent study showed that, in subclinical VK deficiency, PIVKA-II did not correlate with functional protein C levels (20). One possible explanation is that protein C has a more sensitive response to changes in the hepatic VK-dependent carboxylase system (21, 22).

ETP, when measured with thrombomodulin (which accounts for the action of protein C), reflects the action of both the pro- and anticoagulants, mimicking much closer hemostasis as it occurs *in vivo*. Theoretically, patients with IFALD and impaired VK absorption should show lower ETP compared to healthy controls.

Our results indicate that newborns with transient cholestatic jaundice secondary to IFALD receiving oral VK supplementation (by milk fortification or formula) have a similar ETP compared to newborns without IFALD.

There is more than one explanation for the contrasting results between PT/aPTT and thrombin generation in this setting. It could be that, in the short period, the reduced biliary flow did not affect VK absorption. However, previous studies showed that VK absorption was mostly ineffective in these cases, with a rapid increase of circulating PIVKA-II (19).

Another possible explanation lies in the supra-physiological VK concentration, which is a typical feature of preterm infants during the first 2 weeks of life, due to the large amount of VK received at birth and during PN (10). Although VK has limited tissue reserves and is rapidly catabolized, we cannot exclude that thrombin generation is impaired only in the case of prolonged VK deficiency. However, this hypothesis contrasts with the knowledge that functionally deficient plasma factor II is an early indicator of subclinical VK deficiency. Finally, another more mechanistic explanation could be that (unlike PT), ETP especially when measured in the presence of thrombomodulin is responsive to both pro- and anticoagulants. Both are reduced in the investigated condition; therefore, ETP did not show between-group differences.

We should recognize some limitations of this study. Although the ETP with thrombomodulin is a reliable marker of the coagulation process *in vivo*, the relationship among plasma VK, PIVKA-II, and ETP is unknown. We cannot exclude that the ETP fails to detect small differences in VK status. Secondly, this study is a secondary analysis of a limited number of patients without a sample size estimation to account for differences in ETP between IFALD patients and controls. Thirdly, the small number of infants did not allow to take into account confounding factors such as gestational age, antimicrobial therapy and bowel length, which all may influence the VK status.

In conclusion, our data suggest that full enterally-fed patients with IFALD did not express coagulation impairment as assessed by ETP 2 weeks after PN discontinuation. Future studies with adequate sample size should explore the predictivity of ETP in identifying subclinical VK deficiency and the relationship between ETP and PIVKA-II in parenteral-induced neonatal cholestasis.

## Data Availability Statement

The raw data supporting the conclusions of this article will be made available by the authors, without undue reservation.

## Ethics Statement

The studies involving human participants were reviewed and approved by Comitato Etico Milano Area B. Written informed consent to participate in this study was provided by the participants' legal guardian/next of kin.

## Author Contributions

SG was involved in the conception and planning of the study, the acquisition, analysis, and interpretation of data, writing of the first draft, and critical revision of the manuscript. GR contributed to planning of the study, acquisition, analysis, interpretation of data, co-writing, and critical revision of the article. VC was involved in the acquisition and interpretation of the data, and the critical revision of the article. ES was involved in data analysis and critical revision of the article. PR, FP, and FM were involved in the conception of the study, interpretation of the data, and the critical revision of the article. AT was involved in the conception and planning of the study, the interpretation of the data, co-writing, and the critical revision of the article. All authors revised the manuscript, gave final approval of the version to be submitted and agreed to be accountable for all aspects of the work in ensuring that questions related to the accuracy or integrity of any part of the work are appropriately investigated and resolved.

## Conflict of Interest

The authors declare that the research was conducted in the absence of any commercial or financial relationships that could be construed as a potential conflict of interest.

## Abbreviations

APTT: Activated Partial Thromboplastin Time
CRIB II score: Clinical Risk Index for Babies score
ETP: Endogenous Thrombin Potential
IFALD: intestinal failure-associated liver disease
INR: international normalized ratio
NICU: Neonatal Intensive Care Unit
PIVKA: Prothrombin Induced by Vitamin K Absence
PN: Parenteral Nutrition
PT: Prothrombin Time
SGA: Small for Gestational Age
VK: Vitamin K.
